# QTL for microstructural and biophysical muscle properties and body composition in pigs

**DOI:** 10.1186/1471-2156-7-15

**Published:** 2006-03-09

**Authors:** Klaus Wimmers, Ilse Fiedler, Torsten Hardge, Eduard Murani, Karl Schellander, Siriluck Ponsuksili

**Affiliations:** 1 Research Institute for the Biology of Farm Animals (FBN), Research Unit Molecular Biology, 18196 Dummerstorf, Germany; 2 Institute of Animal Science, Animal Breeding and Husbandry Group. University of Bonn, 53115 Bonn, Germany; 3 Research Institute for the Biology of Farm Animals (FBN), Research Unit Growth and Muscle Biology, 18196 Dummerstorf, Germany; 4 Institute of Animal Science, Humboldt University of Berlin, 10115 Berlin, Germany; 5 Research Institute for the Biology of Farm Animals (FBN), Research Group Functional Genomics, 18196 Dummerstorf, Germany; 6 Boehringer Ingelheim

## Abstract

**Background:**

The proportion of muscle fibre types and their size affect muscularity as well as functional properties of the musculature and meat quality. We aimed to identify QTL for microstructural muscle properties including muscle fibre size, their numbers and fibre type proportions as well as biophysical parameters of meat quality and traits related to body composition, i.e. pH, conductivity, area of M. longissimus dorsi and lean meat content. A QTL scan was conducted in a porcine experimental population that is based on Duroc and Berlin Miniature Pig.

**Results:**

Least square regression interval mapping revealed five significant and 42 suggestive QTL for traits related to muscle fibre composition under the line-cross model as well as eight significant and 40 suggestive QTL under the half-sib model. For traits related to body composition and biophysical parameters of meat quality five and twelve significant plus nine and 22 suggestive QTL were found under the line-cross and half-sib model, respectively. Regions with either significant QTL for muscle fibre traits or significant QTL for meat quality and muscularity or both were detected on SSC1, 2, 3, 4, 5, 13, 14, 15, and 16. QTL for microstructural properties explained a larger proportion of variance than did QTL for meat quality and body composition.

**Conclusion:**

Microstructural properties of pig muscle and meat quality are governed by genetic variation at many loci distributed throughout the genome. QTL analysis under both, the line-cross and half-sib model, allows detecting QTL in case of fixation or segregation of the QTL alleles among the founder populations and thus provide comprehensive insight into the genetic variation of the traits under investigation. Genomic regions affecting complex traits of muscularity and meat quality as well as microstructural properties might point to QTL that in first instance affect muscle fibre traits and by this in second instance meat quality. Disentangling complex traits in their constituent phenotypes might facilitate the identification of QTL and the elucidation of the pleiotropic nature of QTL effects.

## Background

The muscular system is the most prominent active part of the locomotor apparatus and accounts for most of the body mass. Muscle tissue largely contributes to the metabolism as well: muscle represents a major site of daily energy consumption, thermoregulation and storage of energy and amino acids. As an important source of proteins for human nutrition, skeletal muscle of farm animals is of interest. Each muscle consists of three main fibre types, slow-twitch oxidative, fast-twitch oxido-glycolytic and fast-twitch glycolytic fibres [1], which are characterised by different microstructural, biochemical and metabolic properties. The number and size of the muscle fibres are major factors determining growth and weight of each muscle. In man, muscle fibre type composition varies considerably due to inherited factors and environmental effects that contribute to phenotypic variation at 45 and 40%, respectively, while the remaining 15% is explained by the error component related to muscle sampling and technical variance [2]. The proportion of type I and II fibres (slow and fast twitch fibres) has been related to insulin resistance and diabetes II predisposition, obesity and body mass index, as well as fat catabolism and capacity to gain or loss weight, i.e. the constitution [3-5]. Muscle fibre composition is to some extent predictive of obesity [6].

Correspondingly, the proportion of muscle fibre types is correlated to growth performance, meatiness and fatness and affects post mortem development of muscle to meat and thus meat quality traits in pigs [7-10]. The rate and extent of post-mortem pH decline is higher in fast-twitch glycolytic muscles with higher glycogen content. Cross sectional area of muscle fibres is negatively correlated to meat tenderness [7-10]. In pig, cattle, mouse, sheep, and horse coefficients of heritability for muscle structure traits were estimated to lie between h^2 ^= 0.20 and 0.60 [10-14].

QTL for muscle fibre traits in pigs were detected on chromosome 8 and chromosomes 1, 2, 6, 14 and X [15,16]. Here we report on the identification of QTL for micro-structural muscle properties, i.e. fibre number, fibre size and fibre type proportions including proportion of defect fibres in a segregating population of pigs derived from Duroc and Berlin Miniature Pig, i.e. breeds being divergent with regard to traits related to growth, muscularity and fatness. Moreover, we identified QTL for traits related to body composition as well as biophysical muscle properties that are technological parameters of meat quality in pig breeding.

## Results

Traits measured and markers genotyped are detailed in Tables 1 and 2. Results of the QTL analyses are compiled in Tables 3 to 6, in which all QTL exceeding the 5% chromosome-wide significance threshold for suggestive linkage are included.

**Table 1:** Means and standard deviations of traits related to muscle fibre size and distribution, muscularity and meat quality measured in M. longissimus dorsi at 13^th^/14^th ^rib on F2 animals of the DUMI resource population

	Trait	Definition	Mean ± SD
1	Fib/mm^2^	mean number of fibres per mm^2^	366 ± 99
	ToF#	total number of fibres at section of M. l. d. [×1000]	803.5 ± 203.5
	Dia_mean_	mean diameter of all fibres [μm]	58.52 ± 7.93
	Dia_AnF_	mean diameter of angular fibres [μm]	23.27 ± 7.09
	Dia_GiF_	mean diameter of giant fibres [μm]	14.05 ± 34.47
	Pro_AnF_	relative proportion of angular fibres [%]	0.90 ± 0.94
	Pro_GiF_	relative proportion of giant fibres [%]	0.05 ± 0.19
	Dia_STO/red_	mean diameter of STO/red fibres [μm]	50.98 ± 7.13
	Dia_FTO/im_	mean diameter of FTO/intermediate fibres [μm]	50.19 ± 8.24
	Dia_FTG/w_	mean diameter of FTG/white fibres [μm]	62.32 ± 9.32
	Pro_STO/red_	relative proportion of STO/red fibres [%]	18.68 ± 6.19
	Pro_FTO/im_	relative proportion of FTO/intermediate fibres [%]	11.34 ± 4.23
	Pro_FTG/w_	relative proportion of FTG/white fibres [%]	69.02 ± 6.49
2	Dia_STO_	mean diameter of STO fibres [μm]	51.60 ± 7.01
	Dia_FTO_	mean diameter of FTO fibres [μm]	49.31 ± 7.44
	Dia_FTG_	mean diameter of FTG fibres [μm]	61.91 ± 9.19
	Pro_STO_	relative proportion of red fibres [%]	15.93 ± 5.40
	Pro_FTO_	relative proportion of intermediate fibres [%]	12.35 ± 4.34
	Pro_FTG_	relative proportion of white fibres [%]	71.34 ± 6.90
	Cap_STO_	mean number of capillaries at STO fibre	1.48 ± 0.46
	Cap_FTO_	mean number of capillaries at FTO fibre	0.96 ± 0.34
	Cap_FTG_	mean number of capillaries at FTG fibre	0.55 ± 0.17
	Cap_mean_	mean number of capillaries at fibres	0.75 ± 0.23
	Cap/mm^2^	mean number of capillaries per mm^2^	82.19 ± 21.86
3	Dia_red_	mean diameter of red fibres [μm]	50.47 ± 7.21
	Dia_im_	mean diameter of intermediate fibres [μm]	50.91 ± 8.81
	Dia_w_	mean diameter of white fibres [μm]	62.66 ± 9.44
	Pro_red_	relative proportion of red fibres [%]	20.95 ± 5.90
	Pro_im_	relative proportion of intermediate fibres [%]	10.50 ± 3.98
	Pro_w_	relative proportion of white fibres [%]	67.11 ± 5.45
4	FOM	Fat-O-Meater: lean meat content according to regression	33.74 ± 8.62
	FOM_M	FOM_meat: depth of M. glutaeus medius	40.01 ± 8.71
	MA_ML_	loin eye area: area of M. l. d. at 13^th^/14^th ^rib [cm^2^]	23.30 ± 4.38
	pH1_ML_	pH1-loin: pH-value 45 min post mortem	6.46 ± 0.25
	pH24_ML_	pH24-loin: pH-value 24 hours post mortem	5.56 ± 0.13
	C1_ML_	conductivity1-loin: conductivity 45 min post mortem [mS/cm]	3.44 ± 0.53
	C24_ML_	conductivity24-loin: conductivity 24 hours post mortem [mS/cm]	3.49 ± 1.22
	MC_Opto_	meat colour, Opto Star: meat colour 24 hours post [%]	69.40 ± 6.29

^1^fibre number and size, fibre type proportions determined by NADH-TR alone and combined NADH-TR/ATPase reaction (n = 308)

^2^fibre size, fibre type proportions determined by combined NADH-TR/ATPase reaction (n = 140)

^3^fibre size, fibre type proportions determined by NADH-TR reaction (n = 168)

^4^carcass and meat quality traits of standard performance test (n = 863) [ZDS]

**Table 2:** Markers used in the QTL analysis and genetic map as established for the DUMI resource population (sex average, Kosambi cM)

Chromosome	Coverage^1 ^[cM]	Markers and genetic distances [cM]								
**SSC1**	16.4 – 140.5 (144.0)	SW1515	33.0	SW1851	33.4	S0155	8.3	RLN*	61.6	SW1301
**SSC2**	0.0 – 74.8^2 ^(132.1)	SW2443	42.7	FTH1*	20.2	SW240	23.0	STS2*	13.8	C3*
					18.3	SW1564	14.2	BHMT*	13.5	S0226
**SSC3**	17.8 – 102.2 (129.3)	SW72	50.8	S0164	25.7	SW2570	33.2	S0002		
**SSC4**	4.1 – 120.0 (130.1)	S0227	47.2	S0001	14.3	STS3*	4.6	CRH*	1.3	STS1*
					1.5	STS4*	28.3	S0214	36.6	S0097
**SSC5**	8.4 – 102.9 (114.4)	SW1482	59.3	SW1134	40.0	IGF1	34.6	SW378		
**SSC6**	7.3 – 102.0 (165.7)^3^	S0035	24.1	HP*	39.4	S0087	10.4	SW1067	9.7	SW193
			5.8	S0300	5.9	TGFB1*	26.9	S0220	35.0	LEP*
							8.5	S0059	16.7	S0003
**SSC7**	3.7 – 134.9 (156.6)	S0025	24.4	S0064	23.5	DQB*	23.5	BF*	15.5	S0102
					15.8	SW175	36.2	S0115	30.2	S0101
**SSC8**	0.0 – 112.3 (127.7)	SW2410	79.5	S0086	24.7	S0144	23.8	SW61		
**SSC9**	11.1 – 96.5 (138.5)	SW21	26.3	SW911	33.1	SW54	17.1	S0109	32.9	S0295
**SSC10**	0. 0 – 124.1 (124.1)	SW830	77.7	S0070	49,3	ITIH2*	36.1	SW2067		
**SSC11**	14.1 – 76.2 (84.9)	SW2008	32.0	S0071	32.0	S0386	34.0	SW703		
**SSC12**	6.6 – 108.3 (113.1)	S0143	49.6	SW874	42.6	SW605				
**SSC13**	1.6 – 79.3 (126.2)	S0219	40.0	SW344	36.6	SW398				
**SSC14**	7.4 – 111.5 (111.5)	SW857	53.4	S0007	30.4	VIN*	37.8	SWC27		
**SSC15**	1.3 – 107.4 (111.8)	S0355	35.3	SW1111	48.5	SW936	33.5	SW1119		
**SSC16**	0.0 – 92.6 (93.2)	S0111	51.2	S0026	42.4	S0061				
**SSC17**	0.0 – 94.0 (97.0)	SW335	34.6	SW840	35.1	SW2431				
**SSC18**	5.0 – 57.6 (57.6)	SW1023	23.9	SW787	43.0	SWR414				

^1^relative position of flanking markers of the set used in the present study on public linkage map (USDA-MARC v2); ^2^S0226 not covered by USDA-MARC v2, but SW14, which is closely linked to S0226 (PigMaP v1.5); ^3^S0035 at 0.0 cM and S0003 at 144.5 cM in the International Workshop 1 SSC6 integrated map with a total length of 166.0 cM; * biallelic markers

**Table 3:** Evidence for QTL significant at the 5% chromosome-wide level for traits related to muscle fibre distribution by chromosome obtained by F2 analysis. Estimated significance levels (F-value), position, % of F2 variance explained by each QTL, and gene effects.

Trait	SSC	Position [cM]	F- Value	% Variance^1^	Additive		Dominance	
					Effect^2^	S.E.	Effect^2^	S.E.
Dia_AnF_	1	3	8.4***	11.8	2.64	1.39	-7.04	1.80
Pro_STO_	1	93	5.5*	10.4	-0.99	1.45	12.97	3.99
Dia_FTG_	1	114	5.0*	9.5	-5.86	2.53	11.77	5.47
Dia_FTG_	2	63	9.4***	17.8	-7.40	1.90	-10.07	5.91
Dia_FTO_	2	63	4.7*	8.8	-4.86	1.68	-4.86	5.06
Dia_mean_	2	66	9.5***	17.9	-7.04	1.78	-8.85	5.58
ToF#	2	141	5.9*	4.9	-78.2	23.7	37.5	37.7
Fib/mm^2^	2	145	6.3*	4.4	-34.0	9.7	67.8	14.3
Pro_GiF_	3	0	6.0*	11.2	0.03	0.02	-0.09	0.03
Fib/mm^2^	3	37	6.9**	3.7	-51.5	14.1	-36.9	27.2
Cap_STO_	3	57	5.7*	10.7	0.07	0.07	0.34	0.11
Pro_FTG_	4	18	6.4*	10.7	-2.47	1.35	-5.76	2.58
Pro_FTG/w_	4	26	7.8**	5.7	-2.98	0.77	-1.00	1.57
Pro_w_	4	33	5.9*	9.3	-2.66	0.97	2.93	1.73
Dia_FTG/w_	4	79	4.9*	3.2	2.38	1.56	5.54	3.04
Fib/mm^2^	4	96	5.4*	10.2	4.11	15.11	-53.30	17.83
Dia_mean_	4	96	7.6**	14.2	0.08	1.89	7.52	2.23
Dia_FTG_	4	96	8.6***	16.3	0.57	2.14	8.82	2.53
Dia_red_	5	0	6.4*	10.2	1.52	2.08	-8.85	3.45
Dia_red_	7	168	5.6*	8.9	2.67	0.93	2.89	1.43
Dia_STO_	9	108	4.3*	8.0	2.84	0.99	0.37	1.36
Cap_mean_	10	54	5.2*	9.8	-0.07	0.05	-0.32	-0.19
Cap_STO_	10	57	6.4**	12.1	-0.21	0.08	-0.49	0.18
Pro_im_	11	21	4.2*	6.7	1.79	0.67	0.84	1.26
Pro_AnF_	11	69	4.9*	9.2	0.01	0.14	-0.90	0.29
Fib/mm^2^	12	90	4.3*	6.8	-40.1	16.9	-40.5	24.6
Dia_red_	12	90	5.6*	8.9	2.89	1.07	3.03	1.57
ToF#	12	92	6.0**	9.7	-92.3	32.8	-92.1	45.1
Dia_STO/red_	12	92	4.6*	3.0	1.85	0.72	1.71	0.98
Pro_red_	14	51	7.3**	11.6	-3.87	1.23	3.11	1.99
Pro_w_	14	54	4.3*	6.6	1.78	1.14	-3.72	1.74
Pro_STO/red_	14	54	6.1*	4.2	-1.99	0.71	1.61	1.20
Fib/mm^2^	14	93	4.9*	7.9	-24.8	28.6	20.0	69.4
ToF#	14	98	7.6**	12.4	-19.9	54.2	461.3	120.6
Dia_im_	14	102	4.2*	6.8	-0.57	1.95	-11.89	4.09
Dia_w_	14	102	7.9***	12.7	1.60	1.96	-15.65	4.09
ToF#	15	56	5.7*	10.8	-98.3	29.0	-7.1	66.9
Pro_AnF_	15	57	5.7*	9.2	0.01	0.22	-1.66	0.51
Dia_GiF_	15	69	5.7**	9.1	21.66	7.11	21.37	14.11
Dia_STO_	15	81	5.4*	10.2	3.15	1.06	-1.98	1.64
Dia_FTO_	15	84	5.8**	11.0	2.70	1.08	-3.48	1.58
Pro_GiF_	16	93	4.5*	8.5	-0.05	0.02	-0.04	0.03
Dia_mean_	18	0	4.5*	8.5	-2.94	1.02	-1.72	1.56
Dia_STO_	18	0	5.6*	10.6	-2.87	0.88	-1.33	1.34
Dia_FTO_	18	0	4.7*	8.9	-2.47	0.96	-2.74	1.47
Dia_STO/red_	18	0	4.8*	3.2	-2.00	0.68	-1.24	0.99
Pro_GiF_	18	48	5.5*	10.4	-0.06	0.03	-0.13	0.05

*: significant at the 5% chromosome-wide level, **: significant at the 1% chromosome-wide level, ***: significant at the 5% genome-wide level

^1^the fraction of phenotypic variance in the F2 explained by a QTL; calculated as the proportion of residual variance of the statistical models with and without the QTL effect

^2^positive values of additive genetic and dominance effects imply higher trait values forced by the Duroc allele;

**Table 4:** Evidence for QTL significant at the 5% chromosome-wide level for traits related to muscle fibre distribution by chromosome obtained by half-sib analysis. Estimated significance levels (F-value), position, % of F2 variance explained by each QTL, and gene effects.

Trait	SSC	Position [cM]	F- Value	% Variance^1^
Pro_GiF_	1	0	8.7*	24.3
Pro_FTG/w_	1	108	4.3**	6.2
Dia_red_	1	117	3.9**	11.0
Fib/mm^2^	1	120	3.4*	9.5
Dia_w_	1	120	4.1**	11.5
Pro_STO/red_	1	124	4.2**	6.0
Pro_red_	1	136	4.5**	12.6
ToF#	2	40	4.8**	15.0
Pro_FTO_	2	100	5.8***	18.2
Cap_STO_	2	123	5.6***	17.5
Pro_im_	3	10	3.3*	9.1
Pro_FTO_	3	16	3.8*	11.9
Dia_Anf_	4	13	4.6**	12.9
Pro_w_	4	19	3.2*	9.0
Pro_FTO/im_	4	64	3.4*	4.8
Pro_FTG/w_	4	80	4.5*	6.4
Dia_FTO/im_	4	80	4.3**	6.2
Dia_GiF_	4	129	4.7*	13.1
Pro_GiF_	4	132	6.2***	8.8
ToF#	5	75	4.3*	12.2
Pro_w_	7	139	3.4*	9.5
Pro_FTO_	8	55	6.0***	18.9
Pro_AnF_	8	97	5.1*	15.9
Pro_FTG_	8	127	5.4**	17.0
Fib/mm^2^	9	50	3.9**	4.1
Dia_FTG/w_	9	72	3.3*	3.2
Pro_AnF_	10	11	4.3*	13.5
ToF#	11	80	6.0**	17.3
Dia_im_	11	83	4.0**	11.1
Dia_red_	11	85	3.9**	11.0
Dia_w_	11	86	4.7**	13.1
Fib/mm^2^	11	91	5.2***	5.9
Pro_red_	11	98	3.5*	9.9
Pro_w_	11	98	3.4*	9.5
Pro_GiF_	12	38	10.3***	24.4
Dia_GiF_	12	56	7.4***	17.5
Cap_mean_	13	54	3.2*	10.0
Dia_FTG/w_	13	60	3.9*	5.6
Fib/mm^2^	13	62	4.1**	5.9
Cap_STO_	13	66	4.0*	12.7
ToF#	13	76	3.2*	9.3
ToF#	14	24	3.9*	12.3
Pro_GiF_	15	32	12.7****	15.5
Dia_GiF_	15	34	6.4**	15.1
Pro_red_	16	12	3.1*	8.6
Pro_GiF_	16	74	4.3*	13.6
Fib/mm^2^	17	22	3.1*	9.7
Pro_AnF_	17	58	4.1**	5.9

*: significant at the 5% chromosome-wide level, **: significant at the 1% chromosome-wide level, ***: significant at the 5% genome-wide level, ****: significant at the 1% genome-wide level

^1^the fraction of phenotypic variance in the F2 explained by a QTL; calculated as the proportion of residual variance of the statistical models with and without the QTL effect

**Table 5:** Evidence for QTL significant at the 5% chromosome-wide level for traits related to muscularity and meat quality by chromosome obtained by F2 analysis. Estimated significance levels (F-value), position, % of F2 variance explained by each QTL, and gene effects.

Trait	SSC	Position [cM]	F-Value	% Variance^1^	Additive		Dominance	
					Effect^2^	S.E.	Effect^2^	S.E.
pH24_ML_	1	21	6.9*	1.8	-0.027	0.007	-0.003	0.013
C1_ML_	2	56	5.88*	1.4	0.146	0.045	0.098	0.118
FOM	3	58	7.9**	2.1	1.66	0.49	1.69	0.78
MC_Opto_	3	0	13.4****	3.6	-1.74	0.35	0.57	0.51
FOM	4	80	19.0****	5.1	3.21	0.53	0.06	0.89
MA_ML_	4	78	13.8****	3.7	1.27	0.25	0.47	0.42
pH24_ML_	4	90	6.4*	1.7	-0.022	0.007	-0.021	0.012
C1_ML_	4	26	5.4*	1.5	0.021	0.039	-0.248	0.076
MA_ML_	6	175	11.4***	3.0	-0.99	0.23	0.52	0.35
C24_ML_	6	138	6.1*	1.5	-0.273	0.083	-0.285	0.184
pH1_ML_	13	76	5.9*	1.6	0.038	0.012	0.021	0.016
pH24_ML_	15	63	5.7*	1.5	-0.031	0.009	0.026	0.024
MC_Opto_	15	117	8.0***	2.2	-1.27	0.37	-0.89	0.50
pH24_ML_	18	66	4.8*	1.3	-0.019	0.007	-0.250	0.012

*: significant at the 5% chromosome-wide level, **: significant at the 1% chromosome-wide level, ***: significant at the 5% genome-wide level, ****: significant at the 1% genome-wide level

^1^the fraction of phenotypic variance in the F2 explained by a QTL; calculated as the proportion of residual variance of the statistical models with and without the QTL effect

^2^positive values of additive genetic and dominance effects imply higher trait values forced by the Duroc allele;

**Table 6:** Evidence for QTL significant at the 5% chromosome-wide level for traits related to muscularity and meat quality by chromosome obtained by half-sib analysis. Estimated significance levels (F-value), position, % of F2 variance explained by each QTL, and gene effects.

Trait	SSC	Position [cM]	F-Value	% Variance
pH1_ML_	1	99	3.5*	2.1
FOM	2	20	6.6****	3.8
MA_ML_	2	20	10.0****	5.8
FOM_M	2	20	2.7*	1.6
C1_ML_	2	24	3.8**	1.8
MC_Opto_	3	0	7.6****	5.6
pH1_ML_	3	24	3.0*	1.7
pH1_ML_	4	12	3.3*	1.9
MA_ML_	4	71	3.2*	1.9
FOM	4	73	5.7****	3.3
MC_Opto_	5	9	6.6***	4.9
FOM	6	0	4.7***	2.7
C24_ML_	6	24	4.6*	2.7
MA_ML_	6	32	5.4****	3.1
MC_Opto_	6	170	4.5*	3.3
pH1_ML_	8	128	3.3*	2.0
MC_Opto_	10	128	2.9*	1.7
C24_ML_	11	36	3.6*	2.1
pH1_ML_	11	72	3.0*	1.7
pH24_ML_	13	0	3.4*	2.0
MC_Opto_	13	43	8.0****	5.9
FOM_M	14	56	2.7*	1.6
C24_ML_	14	120	3.9*	2.2
pH24_ML_	15	48	5.4***	3.1
C1_ML_	15	88	3.0*	1.4
FOM	16	0	5.2****	3.0
FOM_M	16	8	3.8**	2.2
C1_ML_	16	20	4.5***	2.3
MC_Opto_	16	42	4.5*	3.3
MC_Opto_	17	0	3.8*	2.8
C24_ML_	17	0	5.2***	3.0
FOM	17	12	2.7*	1.6
pH1_ML_	17	68	3.1*	1.8
FOM	18	0	3.1*	1.8

*: significant at the 5% chromosome-wide level, **: significant at the 1% chromosome-wide level, ***: significant at the 5% genome-wide level, ****: significant at the 1% genome-wide level

^1^the fraction of phenotypic variance in the F2 explained by a QTL; calculated as the proportion of residual variance of the statistical models with and without the QTL effect

Analysis under the line-cross model assumes that the founder lines were fixed for different QTL alleles. For 30 traits related to muscle fibre composition 47 estimated QTL positions were detected with at least suggestive significance (p < 0.05 for each chromosome, i.e. roughly one false positive per genome). Out of these five reached the genome-wide p <0.05 significance thresholds. For eight traits related to body composition and meat quality 14 computed QTL positions were found with five exceeding the 5% or 1% genome-wide significance threshold. Analysis under the half-sib model does not make the assumption of fixation of QTL alleles in the founder lines. It showed 48 QTL for microstructural muscle properties and 34 for traits of muscularity and biophysical parameters, with 20 reaching genome-wide significance. Estimates of the degree of phenotypic variation explained by the QTL range between some 2% for suggestive QTL for meat quality and carcass traits and 24% for a QTL for proportion of giant fibres on SSC12.

The telomeric region of SSC1 contained a QTL for the diameter of angular fibres (Dia_AnF_) reaching 5% genome-wide significance under the line-cross model. Estimated additive and dominance effects indicated an overdominant QTL (dominance effect > additive genetic effect) with the Miniature Pig allele causing higher trait values (Table 3). The same region showed a suggestive QTL for proportion of giant fibres (Pro_GiF_) under the half-sib model (Table 4). The distal region of SSC1 exhibited a number of suggestive QTL related to the proportion and diameter of slow twitch oxidative (STO) and fast twitch glycolytic (FTG) fibres and fibre number (Tables 3 and 4).

On SSC2 highly significant QTL for meatiness (FOM, MA_ML_) were detected in the proximal region in marker interval SW2443-FTH1 by half-sib analysis (Table 6); according to line-cross model a QTL for mean fibre diameter (Dia_mean_) mapped to the intermediate region of SSC2 (SW240-STS2) that explained a considerable high proportion of phenotypic variation (Table 3); distal of these are QTL with genome-wide significance and strong effects on proportion of slow twitch fibres and their capillarisation (Pro_STO _and Cap_STO_) (C3-SW1564, half-sib model, Table 4).

The telomeric region of SSC3 contained a highly significant QTL for meat colour (MC_Opto_) as detected by half-sib and line-cross analysis (Tables 5, 6). Suggestive QTL for microstructural muscle traits map slightly more distal (Tables 3, 4).

According to the line-cross analysis the intermediate region of SSC4 (STS3-S0214) carried a highly significant QTL for lean meat content, FOM, and eye muscle area, MA_ML _(Table 5) as well as some suggestive QTL for fibre type composition and meat quality (Tables 3, 4, 5). The more proximal area of SSC4 (S0227-S0001) carried suggestive QTL for the proportion of fast twitch fibres (Pro_w_, Pro_FTG_, Pro_FTG/w_), as revealed by the F2 and half-sib model (Tables 3, 4). According to the half-sib model the region between S0214 and S0097 of SSC4 bore QTL for the number and size of giant fibres (Table 4).

Chromosomes 5, 6 and 7 did not show any or just suggestive QTL for microstructural muscle properties. Loci controlling lean meat content segregated on SSC6 in the marker interval S0035-S0087 in the half-sib model and between S0059 and S0003 in the line-cross model (Tables 5, 6). For SSC8 using the half-sib model a significant QTL for proportion of fast twitch oxidative fibres (Pro_FTO_) was detected in the marker interval SW2410-S0086 (Table 4).

While on SSC9 and 10 there were no significant QTL for muscle fibre traits SSC11 had a significant QTL for fibre number per mm^2 ^(Fib/mm^2^) as detected by half-sib analysis between markers S0386 and SW703 (Table 4). According to the half-sib analysis loci affecting proportion and size of giant fibres (Pro_GiF_, Dia_GiF_) segregated on SSC12 close to marker SW874 (Table 4). No QTL reaching genome-wide significance mapped to SSC13.

The distal part of SSC14 (Vin-SWC27) exhibited a significant QTL for fibre diameter (Dia_w_). Dominance effects are larger than additive genetic effects with Duroc alleles decreasing the trait value (Table 3). The same interval harboured a suggestive QTL for meat conductivity (C24_ML_) while the region of SSC14 close to marker S0007 showed suggestive QTL for proportion of different fibre types and FOM shown by line-cross and half sib analysis, respectively (Tables 3, 6).

SSC15 showed significant QTL for proportion and diameter of giant fibres (Pro_GiF_, Dia_GiF_) as revealed by half-sib analysis between markers S0355 and SW1111 as well as suggestive QTL for size and proportion of other fibre types shown by line-cross analysis in interval SW1111-SW936 (Tables 3, 4). Also close to marker SW1111 there was a significant QTL for ph24_ML _detected by the half-sib analysis (Table 6). Even more distal (SW936-SW1119) a significant QTL for meat colour was found in the line cross model analysis (Table 5).

The telomeric regions of SSC16 and SSC17 contained significant QTL for meat quality (C1_ML_, C24_ML_) and carcass traits (FOM) under the half-sib model (Table 6). SSC 18 carried no QTL reaching the genome-wide significance for the traits analysed.

## Discussion

A genome scan was performed in a F2 experimental crossbred population using a marker set covering more than 80% of the porcine linkage map (USDA-MARC v2, ArkDB [17]) for traits related to muscle fibre type composition and meat quality applying line-cross and half-sib analysis. The study showed that microstructural properties of pig muscle as well as meat quality traits are governed by genetic variation at many loci distributed throughout the genome. This study revealed more QTL exceeding the 5% chromosome-wide significant threshold than expected by chance when analysing 38 traits and 18 autosomes. The proportion of phenotypic variation explained was higher for QTL for microstructural properties, ranging between 3.0 and 24.4%, than for meat quality and carcass traits (1.3–5.9%). This indicates higher power of the analyses for the fibre type traits. Muscle fibre type composition is known to affect meat quality with larger fibre diameters and higher proportion of white fibres resulting in poor meat quality [7-10]. Muscle fibre traits have moderate to high heritabilities (h^2 ^= 0.20 – 0.59) compared to meat quality traits (h^2 ^= 0.15 – 0.32) [10]. Thus large effects of QTL for fibre traits might reflect the larger impact of genetic variation on these traits than on meat quality. It should be noticed that the number of animals with muscle fibre phenotypes available for the present study was lower than the number of animals with technological meat quality phenotypes. Therefore the power of the analysis might have not been sufficient to detect even more QTL for fibre traits with lower effects that probably exist. In a genome scan conducted in a resource population based on Japanese Wild Boar and Large White Nii and coworkers (2005) [16] identified QTL for proportion of muscle fibre types and their proportion of relative area of skeletal muscle that explained 5 to 7% of phenotypic variation – similar to QTL for meat colour and pH. The large effects of QTL for fibre traits obtained in the present study may also be due to higher variation in fibre type traits than meat quality traits in the DUMI population. Coefficients of variation for fibre size were close to 15%, for proportion of STO and FTO fibres exceeded 30%, while it was up to 3% for meat pH, 10 and 15% for meat colour and conductivity 45 min post mortem; just coefficient of variation of conductivity 24 hours post mortem exceeded 30%. Coefficient of variation even exceeded 100% for traits related to giant and angular fibres. It should be noticed that angular and giant fibres occurred at a low frequency and thus the traits have distorted distribution, making the estimation of QTL less reliable. The coefficients of variation for fibre type proportion obtained by Nii et al. (2005) [16] were lower (ca. 22%) than in the DUMI population but still higher than coefficients of variation of meat quality traits like pH.

Thus, by looking at the microstructural properties of muscle rather than measuring complex meat quality traits one gets closer to the genes' effects. Moreover, higher phenotypic variation of these traits facilitates the identification of QTL. In consequence, the QTL analysis for both (1) complex traits related to biophysical muscle/meat parameters and (2) microstructural muscle properties – know to be responsible for a large proportion of variation in the aforementioned traits – at the same time, represents a model case that allows dissecting genomic regions, which control these traits, and in which variation in the meat phenotype is probably associated to genes that in first line affect muscle fibre traits.

We found more QTL using the half-sib model than the line-cross model indicating that the founders of the DUMI population are not fixed for different alleles at many of the QTL. In F2 resource populations of Chinese Meishan and commercial pig lines de Koning et al. (2001) [18] as well as Bidanel et al. (2001) [19] also applied line-cross and half-sib models and were able to identify additional QTL due to the use of both. The authors discuss that the use of both models allows investigating different assumptions about QTL genotypes in founder populations that are obviously both true for some QTL in resource populations typically used in farm animals. The line-cross model assuming that different QTL alleles are fixed in founder populations is very powerful when this assumption corresponds to the true state of nature of the QTL and it is quite robust to limited deviations from this ideal situation, even though it tends to underestimate QTL effects in such situations [20]. The half-sib model is more general with no assumption about the number and frequency of QTL alleles in founder populations and probably more realistic for many QTL. This is in line with QTL studies made in commercial pig populations and their crosses showing that even in these selected populations there is still a considerable amount of genetic variation at loci affecting traits of interest [21-23].

In pig breeding biophysical muscle properties like pH and conductivity are measured in routine to monitor meat quality mainly in terms of water binding capacity. At the cellular level, a high proportion of glycolytic muscle fibres, large fibre diameters, low vascularisation and reduced mitochondrial activity are associated with low water binding capacity [24,25]. The present study focuses on these fibre type traits representing cellular factors on meat quality. At the molecular level, pathways involved in energy and calcium homeostasis are of importance. For example, genetic variation of ryanodine receptors, calcium-release channels, has major impact on pH and conductivity in pork and muscle activities such as protein metabolism, differentiation, and growth as well as pathophysiological conditions seen in dystrophinopathies, Brody's disease, and malignant hyperthermia in human [26]. Due to their great economical importance body composition traits, especially lean meat content, are also recorded in routine in pig breeding. Correspondingly, there are many efforts to identify QTL responsible for the variation in technological parameters of meat quality and in body composition traits. Genome scans were conducted in different experimental and commercial populations and revealed QTL effects on all 18 autosomes and chromosome X as currently summarized on the Pig Quantitative Trait Loci (QTL) database (PigQTLdb) [27,28]. Here, we report QTL for meat quality traits with genome-wide significance on SSC3, 5, 13, 15, 16 and 17 and QTL for traits related to lean meat content on SSC2, 4, 6, and 16.

On SSC3 QTL with effect on pH value, meat colour, and juiciness have been found in several experimental populations [18,29-32]. The QTL effects on pH value were validated and confirmed in commercial lines [21,33]. These results together with our data indicate that loci affecting meat colour and meat quality traits related to water binding capacity, like pH value and conductivity, segregate in many populations including commercial breeds and are located on the proximal region of the p-arm of SSC3. Skeletal muscle myosin regulatory light chain 2 (HUMMLC2B) represents a positional functional candidate gene [34]. Malek et al. (2001) [15] detected QTL for meat colour on SSC5, however these map to the distal end close to marker SW378, while the QTL reported in the present study is at the opposite telomer close to marker SW1482. De Koning and co-worker (2001) [18] reported on a QTL for meat colour on SSC13 that corresponds to our finding. On SSC15 effects on pH value and colour matching our QTL have been reported by others [15,18]. Also QTL for glycogen content and/or glycolytic potential were shown [15]. The QTL region comprises PRKAG3, (protein kinase, AMP-activated, y-(3)-subunit), that has been shown to be the causal gene for variation in glycogen content in the muscle and the resulting meat quality [35,36]. To some extent these traits reflect the fibre type distribution for which we found a number of QTL on SSC15. Geldermann et al. (2003) [37] and Paszek et al. (2001) [29] mapped QTL for pH and conductivity on SSC16 in vicinity to those estimated by us. On SSC17 QTL for meat colour, juiciness, lactate concentration, and glycolytic potential were shown by Malek et al. (2001) [15].

The finding of a QTL for muscularity, i.e. FOM and eye muscle area, on SSC2 corresponds to reports of Jeon et al. (1999) [38] and Nezer et al. (1999) [39] on paternally expressed QTL affecting muscle growth. The authors highlighted IGF2 (insulin like growth factor 2) as the positional candidate gene. Subsequent analyses led to the identification of the causal mutation in intron 3 of IGF2 that modulates its expression [40]. Effects of the IGF2 polymorphisms have been confirmed in other populations [41,42] and IGF2 is also a likely candidate for the QTL shown in the present study. The QTL for body composition on SSC4 represent the first ever published in pigs [43]. The QTL have been confirmed in several populations [44]. In a discordant sibpair analysis using AFLP markers we also identified QTL for body composition traits on SSC4 and proposed CRH (corticotropin releasing hormone) gene as a functional positional candidate gene [45]. QTL for muscularity have been previously shown in the central and distal region of SSC6 [30,46], however the QTL for muscularity proximal on SSC6 shown in the present study are new, as well as the QTL for lean meat content on SSC16.

Recently, Nii and coworkers (2005) [16] report on QTL for muscle fibre traits and meat quality in a Japanese Wild Boar × Large White intercross on SSC 1, 2, 6, 14, and X. Discrimination of type I, IIA, or IIB fibres was based on myosine ATP method after alkaline preincubation expected to reveal phenotypes corresponding to ours. Nii and co-worker (2005) [16] found genome-wide significant QTL for type I fibres on SSC1 and 14 in vicinity of the regions where we found chromosome-wide significant QTL for slow twitch and red fibres, respectively. According to Nii et al. [16], positional candidate genes for the QTL on SSC14 are genes of the calcineurin signalling pathway that is involved in muscle fibre type switch, namely PPP3CC (protein phosphatase 3 catalytic subunit γ isoform, calcineurin A γ), PPP3CB (protein phosphatase 3 catalytic subunit β isoform, calcineurin A β) and NFAM1 (NFAT activation module 1). Moreover, QTL for type IIA, IIB and intermediate, white, and fast twitch fibres on the intermediate region of SSC2 and proximal on SSC14 have been found by us and Nii (2005) [16]. QTL for proportion of type I fibres were detected on SSC8.

In mice genome-wide significant QTL for weight of muscles with different muscle fibre type composition were detected on chromosome 3, 8, and 17 that were muscle-specific but not muscle fibre type specific [47]. According to the actual comparative map, these regions correspond to the proximal part of SSC6, q-arm of SSC14 and central region of SSC7. In that region of SSC6 we did not detect QTL for muscle fibre composition but for FOM and MA_ML_.

Thus the results correspond in indicating QTL for size of particular muscle, M. gastrognemius in mice and M. longissimus dorsi in pig, however the first is a mixed fiber muscle while the second consists mainly of fast fibres. The mouse QTL on chromosome 8 affects size of the fast muscle extensor digitorum. QTL in the corresponding porcine genome regions are for fibre number and diameter. We found no significant QTL on SSC7. Studies of the potential role of the myostatin pathway on muscle strength in human revealed linkage of markers close to myostatin (GDF8) on HSA2, to CDKN1A (cyclin-dependent kinase inhibitor 1A) on HSA6, and to myogenic factor 3 (MYOD1) on HSA11, as well as markers on HSA12 and HSA13 [48,49] to potential QTL. In cattle and mouse myostatin has been shown to affect muscle mass [50,51]. Porcine myostatin maps to SSC15q23 [52] and it is a positional functional candidate gene for the QTL for muscle fibre type traits and biophysical muscle properties identified here. The QTL regions on HSA6, 11, 12, and 13 correspond to the proximal half of SSC7, p-arm of SSC2 (MYOD1 at 2p14-17; [53]), SSC 5, and SSC11. We found QTL for fibre diameters and fibres number on SSC2 and 11. Genes of the myostatin pathway are functional candidates for traits related to muscle strength and dynamic properties as well as fibre composition traits in man and pig. Other established functional candidate genes for muscle fibre traits that represent positional candidates for the QTL detected in this study [54,55], i.e. MEF2C (MADS box transcription enhancer factor 2, polypeptide C; SSC2), MEF2D (MADS box transcription enhancer factor 2, polypeptide D; SSC4), PPARGC1A, (peroxisome proliferative activated receptor, gamma, coactivator 1, alpha; SSC8) and PPARG (peroxisome proliferator-activated receptor gamma 2; SSC13).

In particular, those genomic regions are of interest that exhibit both (1) QTL for body composition and biophysical muscle properties, which are used as technological parameters of meat quality to select in breeding routine, and (2) QTL for muscle fibre number and distribution traits, which are more strictly genetically controlled but affect growth, body composition and meat quality to a large extent.

**Figure 1:**
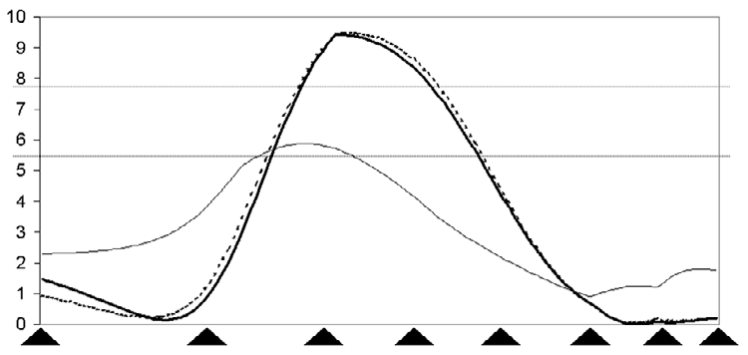
Plot of the F-ratio from least square interval mapping for evidence of QTL for DIA_FTG _() and DIA_mean _() as well as C1_ML _() on SSC2. The x-axis indicates the relative position on the linkage map. The y-axis represents the F-value. Arrows on the x-axis indicate the position of markers (SW2443, FTH1, SW240, STS2, C3, SW1564, BHMT, S0226). Lines indicate the 5% genome-wide and 5% chromosome-wide significance thresholds.

Taking into account QTL with genome-wide significance we found correspondence between the QTL for pH24_ML _and the QTL for Pro_GiF _on SSC15. It should be noticed that giant fibres occurred at a low frequency and thus the trait has distorted distribution, making the estimation of QTL less reliable. Taking a further look at regions with either genome-wide significant QTL for fibre type traits or genome-wide significant QTL for meat quality reveals further correspondence on SSC1 (pH24_ML _and Dia_AnF_), on SSC2 (C1_ML _and Dia_mean _and Dia_FTG_; Figure 1), on SSC3 (MC_opto _and Pro_Gif_, Pro_im_, Pro_FTO_), on SSC5 (MC_opto _and Dia_red_), on SSC13 (MC_opto _and Fib/mm^2^), on SSC14 (C24_ML _and Dia_w_), and on SSC16 (C1_ML _and Pro_red_). Looking at all QTL detected even more regions appear with QTL for fibre type distribution traits and meat quality traits on SSC4, 8, 11, and 17. With regard to the relationship between meat quality, lean meat content and fibre type distribution traits p-arm of SSC2 and proximal region of q-arm of SSC4 are of interest. On SSC2 and SSC4 we found genome-wide significant QTL for FOM and area of M longissimus on the one hand and fibre diameter (Dia_FTG_, Dia_mean_) on the other hand that might again indicate a common genetic background. Also on SSC14, 16, 17, and 18 we found QTL for body composition and muscle fibre traits. On SSC7 and 9 we only found QTL for muscle fibre type distribution traits. SSC6 showed genome-wide significant QTL for FOM and MA_ML _and for meat quality traits, but no QTL for muscle fibre traits. Nii et al. (2005) [16] found QTL for meat colour and hematin content on SSC6 close to QTL for type II fibres.

## Conclusion

Microstructural properties of pig muscle and meat quality are governed by genetic variation at many loci distributed throughout the genome. The number and type of muscle fibres affect body composition and muscle properties and are to a large extent determined prenatal by genetic factors. The application of linkage analyses, which are a priori hypothesis-free, on traits of high heritability increases the power of the approach. Disentangling complex traits in their constituent phenotypes might facilitate the identification of QTL and the elucidation of the pleiotropic nature of QTL effects. QTL analysis under both, the line-cross and half-sib model, allows detecting QTL that are fixed or segregating among the founder populations and thus provide comprehensive insight into the genetic variation of the traits under investigation. The map-based data provided here will facilitate the identification of genes directly affecting muscle fibre traits and indirectly meat quality traits especially when combined together with current attempts to identify genes expressed during myogenesis [56,57]. In those regions harbouring QTL for muscle fibre traits and QTL for meat quality and body composition traits, i.e. on SSC1, SSC15 (with QTL for pH and size and proportion of defect fibres), on SSC2, SSC4 and on SSC14 (with QTL for conductivity, muscularity and fibre size) and on SSC3 (with QTL for meat colour and fibre proportion) effects on meat quality traits and body composition might be the result of genetic variation primarily affecting muscle fibre traits.

## Methods

### Animals

Analyses were done in an experimental population based on reciprocal crossbreeding of Duroc and Berlin Miniature Pig breeds that is a three-generation porcine F2 population (DUMI population). In detail, a Duroc boar was mated to four Berlin Miniature Pig dams and a Berlin Miniature Pig boar was mated to five Duroc dams to produce 43 F1 dams and 5 F1 boars. F1 boars and dams were kept at two places to finally produce 905 F2-animals in total. Thirty-three full sib-families comprising 469 F2-individuals born from 32 sows and four boars were kept and performance tested on the research farm of the Institute of Animal Science in Berlin Dahlem, Humboldt University of Berlin (up to day 100) and at the performance test station of the federal country Brandenburg (day 100 to day 200). At the research farm Frankenforst of the Institute of Animal Breeding and Genetics, University of Bonn, 436 F2 animals of 21 full sib-families were born from 11 sows and three boars, raised and performance tested. F2 piglets were weaned at about 6 weeks of age and kept in flat decks until day 100 and subsequently in single pens until slaughter at 200 days of age. The F2-animals of the DUMI population have obese carcasses with lean meat content of about 34% (FOM). Mean daily gain (weight gain during performance test; day 110 to 200) was 470 ± 117 g with weight at day 200 ranging between 29 and 104 kg [58].

### Phenotypes

Traits related to body composition as well as biophysical muscle properties are defined and listed in Table 1 together with their mean values. Correlations between muscle fibre traits and meat quality ranged between 0.1 and 0.3 [58]. Muscle fibre characteristics of the longissimus muscle were determined by microscopic image analyses after histochemical fibre type differentiation. The samples were taken immediately post mortem at the 13^th^/14^th ^rib, frozen in liquid nitrogen and stored at -70°C. Serial cross-sections (12 μm) were obtained in a cryostat microtom (-20°C) in order to be processed for the following histochemical reactions:

The definitions of muscle fibre distribution traits as well as their mean values are given in Table 5. In order to differentiate the three main fibre types "red", "intermediate", "white" and "slow twitch oxidative = STO", "fast twitch oxidative = FTO", "fast twitch glycolytic = FTG", respectively, the samples were stained either by the NADH tetrazolium reductase reaction (NADH-TR) alone or by the combined NADH-TR/ATPase reaction described by Horak (1983) [59]. While using just the NADH-TR reaction allows differentiating fibres according to their high, moderate or low oxidative enzyme activity, the combined method of NADH-TR and acid stable myofibrillar ATPase reaction indicates the contractile properties of the fast or slow fibre type, respectively [60]. In addition, the hyper-contracted giant fibres were classified by their typical oval or round shape and/or their large size independent of their specific enzyme activity [61]. Giant fibres display a condition where single fibre loose their connection within the muscle tissue and therefore contract maximal. Occurrence of giant fibres is correlated with inferior meat quality [58,61]. The angular fibres were classified and measured based on their characteristic right-angled shape with concave borders, described by Walasik et al. (2000) [62].

For the identification of the capillaries the alkaline phosphatase reaction was used detecting this marker enzyme of endothelial cells as described by Josza et al. (1993) [63].

**Figure 2:**
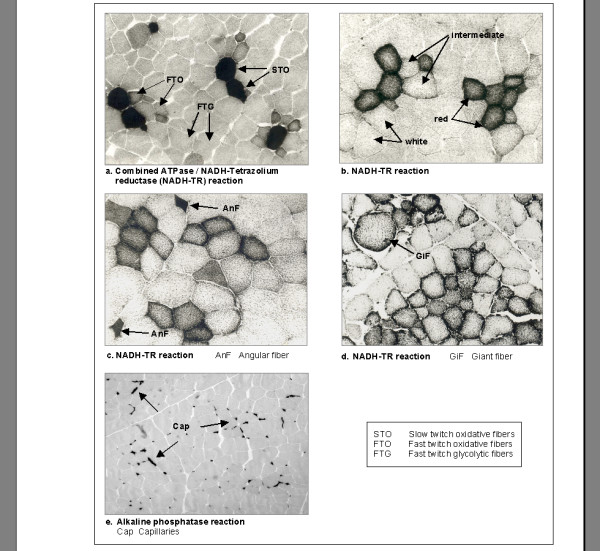
Cross sections of fibres in M. longissimus dorsi. Combined ATPase/NADH-TR reaction was used to identify STO, FTO and FTG fibres (a) while NADH-TR reaction was used to identify red, intermediate and white fibres as well as angular and giant fibres (b, c, d). For the identification of the capillaries the alkaline phosphatase reaction was used (e). Magnification: 200× (a-d); 100× (e).

The staining results of the fibre type classification by the NADH-TR reaction and by the combined NADH-TR/ATPase reactions and the identification of the capillaries, giant fibres, and angular fibres are shown in Figure 2.

The quantitative microscopic determination of fibre type proportion, fibre size and capillar density was done on 400 fibre cross sections per animal by using the image analysis systems AMBA (IBSB, Berlin, Germany) [64] and semiautomatic computer-aided image analyser MFA (muscle fibre analyser) [65]. Taking into account of the special structure of the longissimus muscle and the clustered slow twitch fibres, all fibres of the primary bundles, which were completely visible, were studied. These bundles were randomly selected over the slides.

In total 308 F2 animals were examined for microstructural muscle traits. QTL analysis for these traits involving differentiation of fibre types based on staining intensity was performed separately for the two subsets of the material with phenotypic evaluation using either NADH-TR (n = 168) or combined NADH-TR/ATPase reaction (n = 140). In the pig muscle fibre typings based solely on metabolic properties (NADH-TR) or combined determination of metabolic and myofibrillar ATPase stability are highly corresponding [66]. Therefore QTL analysis was also performed for the whole data set with traits named with the suffix "STO/red", "FTO/im" and "FTG/w".

### Markers and QTL-analysis

Altogether the animals of the DUMI population were genotyped at 88 loci covering the porcine autosomes with mean interval size of 30.7 cM. The set of markers includes 72 microsatellites and 16 biallelic markers. Linkage analysis was performed using the program CRI MAP, version 2.4 [67]. The order of markers and the genetic distances between them are given in Table 2.

The QTL analysis was done with the program QTL express accessible via internet [68,69] by interval mapping based on least square regression analysis developed for three generation F2 populations and half-sib families [70,71]. Additive genetic effects were estimated at 1 cM intervals as half of the difference of the trait value between homozygous carriers of the Duroc and the Miniature Pig alleles; i.e. positive values for the additive of genetic effects point to a higher trait value for homozygous carriers of the Duroc allele. Dominance effects are estimated as the difference between the trait value of heterozygous individuals and the mean trait value observed for homozygous animals. Subsequently, additive and dominant coefficients at fixed positions in the genome of each F2 animal and their phenotypic values were regressed onto the additive and dominance coefficients in intervals of 1 cM. Least square regression models used for QTL analysis included along with additive and dominance coefficients for the putative QTL the fixed effects of family, parity and sex as well as slaughter weight as a co-variable, which were found to affect almost all traits analyses in previous analyses of variance ignoring any molecular genetic information.

Also a paternal half-sib analysis was accomplished making no assumptions on the relative frequencies of the QTL alleles in the founder populations. Therefore the F2-animals were treated as paternal half-sib families and the probability for the occurrence of a paternal allele was estimated in intervals of 1 cM. The probabilities of inheritance of distinct paternal gametic phases were regressed onto allele substitution effects at the putative QTL. The regression model included sex and litter as fixed effects and slaughter weight as co variable.

Significance thresholds at the 5 and 1% level were determined empirically by permutation for individual chromosomes [72]. Chromosome-wide 1 and 5% significance thresholds became genome-wide significance thresholds after Bonferroni correction for 18 autosomes of the haploid porcine genome.

## Authors' contributions

KW significantly contributed to the concept and design of the study and data analysis and wrote the manuscript. IF performed phenotypic analyses and helped in drafting the manuscript. TH was largely involved in design and coordination of the study. EM carried out genotyping and assisted statistical analysis and preparing the manuscript. KS made substantial contribution to the design of the study, coordination of data collection and helped in drafting the manuscript. SP provided input to design of the study, performed molecular genetic and statistical analyses and assisted drafting the manuscript and revising it critically for scientific content.
